# Scaling laws explain foraminiferal pore patterns

**DOI:** 10.1038/s41598-019-45617-x

**Published:** 2019-06-24

**Authors:** Julien Richirt, Stéphane Champmartin, Magali Schweizer, Aurélia Mouret, Jassin Petersen, Abdelhak Ambari, Frans J. Jorissen

**Affiliations:** 1 UMR 6112 LPG-BIAF Recent and Fossil Bio-Indicators, Angers University, 2 Bd Lavoisier, F-49045 Angers, France; 2 LAMPA, Arts et Métiers ParisTech, 2 Bd du Ronceray, BP 93525, 49035, Angers, Cedex 01 France; 3 Institute of Geology and Mineralogy, University of Cologne, Zülpicher Str. 49a, 50674 Cologne, Germany

**Keywords:** Marine biology, Palaeoceanography

## Abstract

Due to climate warming and increased anthropogenic impact, a decrease of ocean water oxygenation is expected in the near future, with major consequences for marine life. In this context, it is essential to develop reliable tools to assess past oxygen concentrations in the ocean, to better forecast these future changes. Recently, foraminiferal pore patterns have been proposed as a bottom water oxygenation proxy, but the parameters controlling foraminiferal pore patterns are still largely unknown. Here we use scaling laws to describe how both gas exchanges (metabolic needs) and mechanical constraints (shell robustness) control foraminiferal pore patterns. The derived mathematical model shows that only specific combinations of pore density and size are physically feasible. Maximum porosity, of about 30%, can only be obtained by simultaneously increasing pore size and decreasing pore density. A large empirical data set of pore data obtained for three pseudocryptic phylotypes of *Ammonia*, a common intertidal genus from the eastern Atlantic, strongly supports this conclusion. These new findings provide basic mechanistic understanding of the complex controls of foraminiferal pore patterns and give a solid starting point for the development of proxies of past oxygen concentrations based on these morphological features. Pore size and pore density are largely interdependent, and both have to be considered when describing pore patterns.

## Introduction

Marine foraminifera are unicellular eukaryotes inhabiting both the benthic and the pelagic realms. They are one of the most widespread groups of marine organisms, constitute the most diverse group of shelled microorganisms in the modern ocean and have a very rich fossil record^1,2^. Foraminifera have been intensively used in paleoceanographic studies and most of our knowledge of the response of past oceans to climate change has been obtained through geochemical measurements of foraminiferal tests^3^. Recently, porosity in benthic foraminifera has been proposed as a proxy of past bottom water oxygen^4–7^ and nitrate levels^8,9^. In view of the expected future decrease in marine oxygen levels, due to global warming and increased eutrophication^10–12^, a precise knowledge of oxygen levels in the past, under different climate regimes, is crucial.

Pores are important morphological features in hyaline foraminifera, but their process of formation and their functions are still very poorly known. Different functionalities have been proposed for these connexions between the cell and the surrounding environment, such as passages for pseudopods^13–15^, buoyancy control^16^ (i.e. in planktonic species), expulsion of gametes^16^, osmoregulation^17,18^, feeding^17^ (intake of organic soluble substances, e. g. dissolved amino acids in sea water) or gas exchanges^17,19–22^. Foraminiferal pores show a large variability in form, size and density. The overall porosity (i.e. the percentage of the test surface covered by pores), which is determined by the latter three factors, is an integrative parameter and studying its variability in relation to environmental parameters may help to understand the functions of pores. In fact, changes in overall porosity can be explained in two ways: (1) as a phenotypic adaptation to external (environmental) parameters, such as temperature, oxygen or nitrate concentration^8,23,24^, or (2) as an internal, species specific, evolutionary adaptation of the genome^25^. In both cases, the physiology of the organism (e.g. metabolic processes) will be modified^18,20,21^.

In order to cope with low oxygen concentrations, benthic foraminifera have developed a range of mechanisms such as nitrate respiration^8,26,27^, sequestration of chloroplasts^28,29^, bacterial symbionts^21^, ultrastructural adaptations^20,21^ or dormancy^30,31^. However, *Ammonia* was shown unable to sequester chloroplasts^29^ and seems strictly aerobic^31^. Intensifying gas exchanges by increasing overall porosity could be another adaptation to hypoxia.

In fact, recently, the variability of pore patterns in benthic foraminifera has been increasingly attributed to differences in gas exchanges, in particular the uptake of oxygen from the surrounding sediment pore waters. The overarching idea is that, when dealing with low oxygen concentrations, a higher total porosity would allow foraminifera to increase their oxygen uptake^20,21,24,32–35^. In several studies, a correlation has been observed between the pore density (number of pores per unit of surface) and the concentration of dissolved oxygen in the surrounding waters^4,5,23^. In these studies, the authors showed that the pore density increased with lower dissolved oxygen concentrations in the surrounding water. Evidently, not only the pore density and the pore size, but also the test thickness will determine the intensity of exchanges through pores. Numerous authors already noted that thin walled species (i.e. with faster gas exchanges) strongly dominate foraminiferal assemblages in oxygen-depleted environments^33,36–38^. Finally, mechanical constraints are necessarily involved when foraminifera adapt their porosity in function of the environment. Foraminifera cannot indefinitely increase the porosity or decrease the thickness of their tests without substantially diminishing the test robustness.

Here we present a simple physical model predicting the relationship between shell porosity, metabolic needs and test robustness, for the last formed chamber of the foraminiferal test. The proposed scaling law model is built on two main assumptions: (1) overall foraminiferal porosity reflects the intensity of gas exchanges, which is determined by cell volume and gas concentrations in the surrounding seawater, and (2) there is a mechanical constraint (test robustness) that limits the increase in overall porosity. Greater porosity can essentially be achieved by increasing pore density and/or pore size. We will use a scaling law model to investigate what range of combinations of these two parameters is physically possible, and what is the optimal response to the combined mechanical and respiratory constraints. We focus on the last formed chamber, which contains the largest volume of protoplasm and is the thinnest one, so that exchanges with the environment are maximal and test robustness is most critical. An important additional reason to use the last formed chamber is that in the foraminifera with lamellar test studied here, a thin calcite layer is precipitated over the entire test with every new chamber. Although most of the pores remain functional, these secondary calcite layers may cause slight changes of the pore characteristics of earlier chambers. Consequently, only the last chamber is fully representative of the trade-off between gas exchanges and test robustness at the time of chamber formation. Finally, we will use a large empirical data set obtained for three phylotypes of the coastal genus *Ammonia*, with very different pore patterns, to verify whether the scaling law model results correctly predict the pore patterns we observed in nature.

## Results

### The model

In order to keep the manuscript as concise as possible, we only present in this section the main features of our model which is developed in detail at the end of the manuscript (see Methods section). The theoretical scaling law model is based on two constraints: the foraminifer needs a minimal respiration rate in order to ensure a nominal metabolism (i.e. “metabolic constraint”) and the mechanical resistance of the shell has to be guaranteed (i.e. “mechanical constraint”).

**Figure 1:**
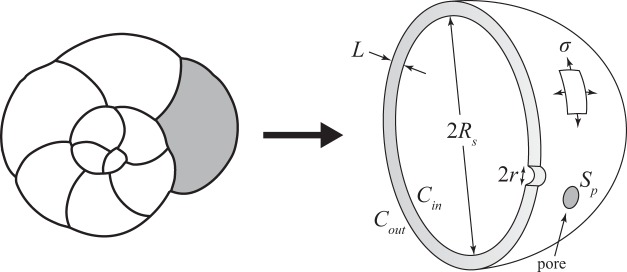
On the left a sketch representing an *Ammonia* sp. specimen in spiral view with the last chamber in grey. On the right, a detailed scheme of the last chamber illustrating the theoretical model. *L*: thickness of the test; *R*_*s*_: radius of the last chamber; *C*_*in*_: gas concentration within the cell; *C*_*out*_: gas concentration in the surrounding water; *r*: mean radius of the pores; *S*_*p*_: mean surface of the pores; *σ*: mechanical stress.

Considering the micrometric size of the pores, passive diffusion controls the transport of gas across the shell. In case of lower oxygen concentrations, the difference of gas concentrations between the foraminiferal cell (*C*_*in*_, Fig. 1) and the surrounding sea water (*C*_*out*_, Fig. 1) will decrease, which according to Fick’s first law of diffusion, will lead to a decrease in the mass transfer through the pores. Here we assumed that the cell adapts its morphological features (i.e. pore patterns) to maintain a constant metabolic rate. In order to compensate for this mass transfer decrease, the test porosity must increase. This can be achieved by (1) an increase in pore density *N*, (2) an increase in pore size (radius *r*), or (3) an increase of both parameters.

**Figure 2:**
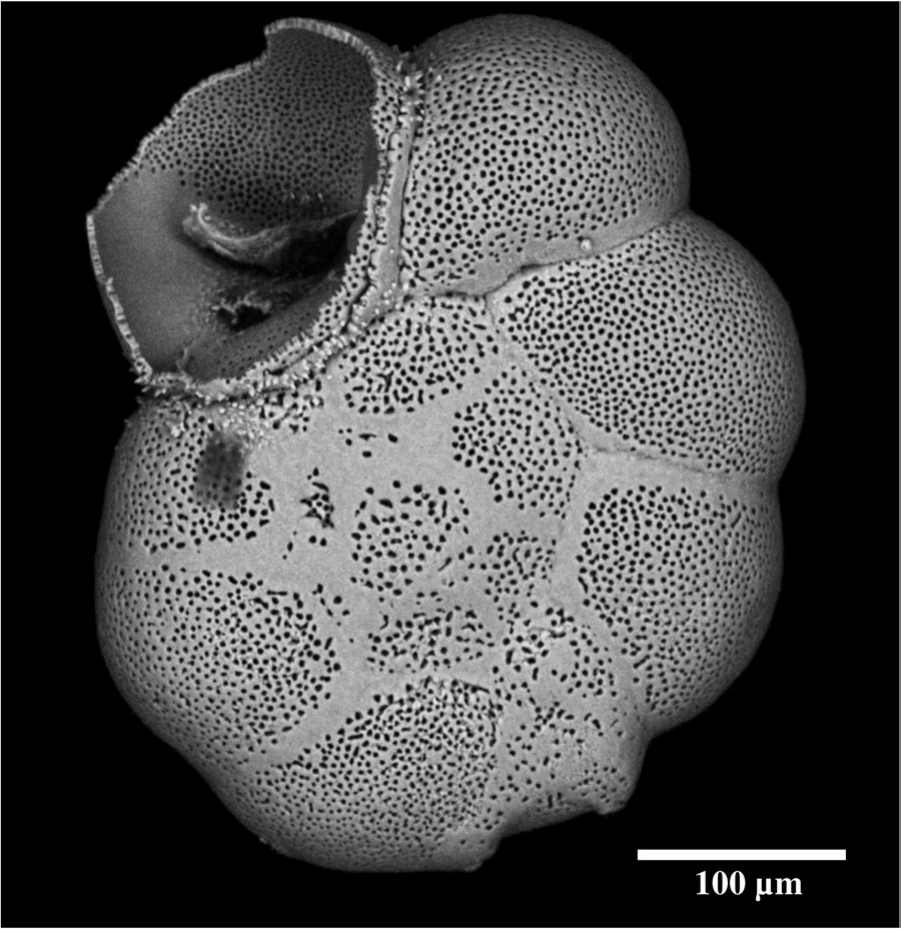
SEM picture of an *Ammonia* sp. specimen showing the response of the foraminiferal test to strong mechanical pressure (notice the net fracture on the last chamber).

As shown on Fig. 1, we considered the individual test chamber as a spherical shell, of radius *R*_*s*_ and thickness *L* ≪ *R*_*s*_. Since the test is mainly composed of calcite, we expect that only little plastic deformation is possible, and that in case of increasing mechanical stress, brittle fractures will rapidly occur, ultimately leading to breakage of the test. (see Fig. 2). The failure theory predicts that this rupture will begin at a point of maximal stress occurring at geometric discontinuities. In the present case, we assume that the pores behave like such stress concentration points. From a mechanical point of view, a fracture may happen if the stress magnitude *σ* in the shell exceeds a limit σ*, the latter being related to the pore structure.

The theoretical scaling law model presented here shows that the three basic ways to increase test porosity have very different consequences from a mechanical point of view. As detailed in the Methods section, the relationships between pore density *N*, pore radius *r*, porosity Φ and the mechanical stress at which test failure occurs σ* can be defined as:

1 $$N \sim \frac{1}{{r}^{3/2}}$$

2 $${\rm{\Phi }} \sim {r}^{1/2} \sim \frac{1}{{N}^{1/3}}$$

3 $${\sigma }^{\ast } \sim \frac{1}{{r}^{1/2}} \sim {N}^{1/3}$$

We can compare these scaling laws with the results of an another simple theoretical approach, a mathematical optimisation of the three parameters above (see Methods), which gives:

4 $$N \sim \frac{1}{{r}^{5/4}}$$

5 $${\rm{\Phi }} \sim {r}^{3/4}$$

### Comparison with an empirical data set

The 1386 individuals used in this study were sampled at 36 different intertidal and subtidal locations with weak hydrodynamics, mainly along the French Atlantic and Dutch coasts (see Methods). The specimens investigated originate from living natural populations (80%), subrecent fossil samples (15%) and living specimens used in laboratory experiments (5%). The measures of the pore density *N*, mean pore radius *r* and porosity Φ were achieved following the methodology developed by Petersen *et al*.^35^. Since in our recent specimens the last chamber is very often broken, we systematically measured pore patterns in the penultimate chamber. The range of pore radii measured on the spiral face (SEM images) shows that the three *Ammonia* sp. phylotypes T1, T2 and T6^39,40^ are mixed in the present data set^41^.

**Figure 3:**
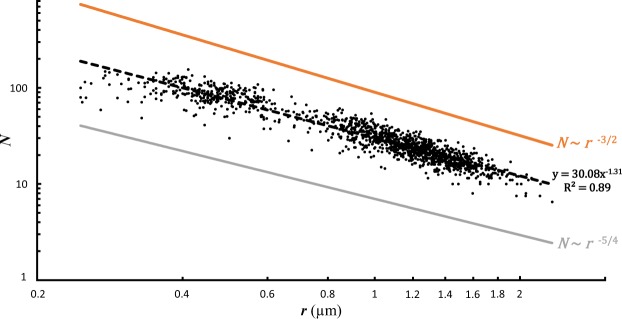
Pore density (*N*, number of pores per 562 µm²) as a function of the mean radius of the pores (*r* in µm). The black dots represent the empirical data. The black dotted line represents the power law model based on the empirical data (y = 30.08 ± 1.01x^−1.31±0.01^, p-value < 2^−16^). The orange line represents the mathematical rule derived from the scaling law model (*N* ~ *r*^−3/2^). The grey line represents the mathematical rule derived from the mathematical optimisation (*N* ~ *r*^−5/4^).

**Figure 4:**
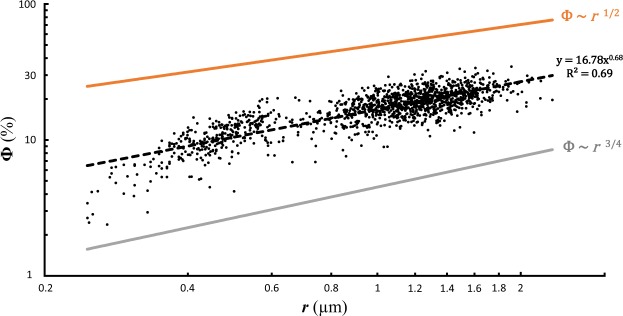
Overall porosity (Ф in % of total test surface) as a function of the mean radius of the pores (*r* in µm). The black dots represent the empirical data, the black dotted line represents the power law model based on the empirical data (y = 16.78 ± 1.01 × ^0.68±0.01^, p-value < 2^−16^) and the orange line represents the mathematical rule derived from the scaling law model (Ф ~ *r*^1/2^). The grey line represents the mathematical rule derived from the mathematical optimisation (Ф ~ *r*^3/4^).

**Figure 5:**
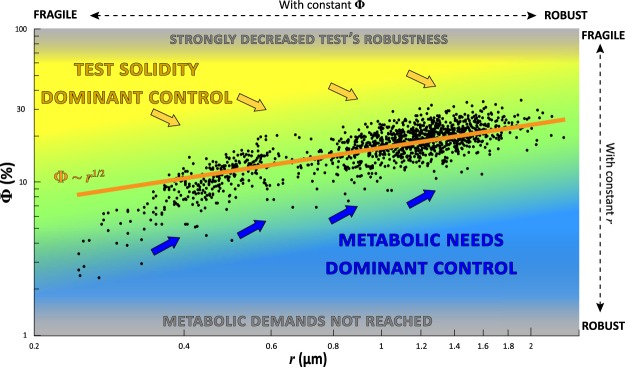
Overall test porosity as a function of pore radius in the empirical data set (black dots, n = 1386), compared with the results of the scaling law model (the orange line Ф ~ *r*^1/2^). In the central part of the graph, there is an “equilibrium area” (in green) where the very large majority of individuals are plotting, in total agreement with the scaling law model. The blue area represents combinations of pore density and radius which are not encountered in nature because the metabolic demands are not fulfilled. Similarly, the yellow area represents the space where the mechanical constraints on the test are too high. The blue arrows represent the direction of the constraints imposed by the need of increased gas exchanges (toward higher porosity and increased pore radius). The yellow arrows represent the direction of the constraints imposed by the mechanical solidity of the test (toward lower porosity and larger pore radius).

Figure 3 displays the empirical data obtained for the pore density *N* (number of pores per 562 µm²) and mean pore radius *r* (in µm) together with the allometric scaling. The theoretical scaling law model and the mathematical optimisation approach only predict the exponent and not the intersect. Therefore, on the Figs 3–5, the lines representing the model outcomes can be moved vertically in an arbitrary way, and we choose the offsets in the graphics in order to avoid superposition with empirical data to make the figures more readable. The empirical observations show a strong relationship between these two parameters described by $N \sim {r}^{-1.31}$. This value is intermediate between the coefficient of −1.5 provided by the model (Eq. 1) and −1.25 given by the mathematical optimisation (Eq. 4).

Figure 4 shows the empirical data for total porosity using the best allometric scaling. The empirical data scale as ${\rm{\Phi }} \sim {r}^{0.68}$; which is intermediate between the exponent of 0.5 predicted by the model (Eq. 2) and 0.75, given by the mathematical optimisation (Eq. 5). Again, the model and the observed data are in close agreement, given our simplified approach and uncertainties in natural settings. Similarly to what is shown in Fig. 3, porosity as observed in the empirical data set increases slightly faster with *r* than the predictions of the scaling law model. In fact, such minor deviations from the predicted exponents are generally interpreted as variations of the system dynamics, which can only be better understood by more exhaustive investigations.

Remarkably, the model predicts that, as a result of mechanical constraint, an increase in total porosity is achieved by a concomitant increase in the pore radius *r* and a decrease in the pore number *N*. This somewhat counterintuitive finding (increased porosity is obtained by simultaneous changes with an opposed individual effect) is fully confirmed by the empirical data. There are several reasons for this. First of all, the impact of pore density and pore size on total porosity is not equivalent: since Ф is proportional to *N* and to *r*², porosity is much more sensitive to a change in *r* than in *N*. Concerning the decrease in *N* in the model results, the explanation is provided by mechanical constraints. In fact, pores act as defects, which may be the origin of fractures or shell breakage (Fig. 2). The lower the pore density is, the less likely test failure becomes. According to the theoretical model, when the pore radius grows to allow increased gas exchanges, a decrease in pore density allows preserving the same mechanical resistance of the test, with a larger overall porosity. However, according to Eq. 3, an increase in the pore radius will also weaken the mechanical resistance of the test, because the stress σ* at which test failure occurs decreases. In fact, the response of the limit stress σ* is more sensitive to variations in pore density *N* than in pore radius *r*.

Summarizing, the model results conclusively show that in order to increase porosity and maintain the mechanical integrity of the test at the same time, the only viable strategy is to increase the pore radius and concomitantly decrease the pore density. It is remarkable that the empirical data set shows exactly the same pattern, strongly confirming the general outcome of the scaling law model.

## Discussion

### Mechanical constraints as physical control of pore patterns

Based on two simple assumptions (i.e. total porosity is controlled by metabolic demands and mechanical constraints), the obtained scaling law model fits surprisingly well with the large empirical data set of measured pore patterns. It is especially satisfactory that the counterintuitive outcome of the model, i.e. that increased porosity can best be achieved by concomitantly increasing the pore radius and decreasing the pore density, is fully confirmed by the empirical dataset. This dual result underlines the complex relationship between pore radius, pore density and overall porosity, and shows that only a limited number of combinations can be realised in nature (Fig. 5).

Foraminiferal test porosity has long been considered as the critical parameter for gas exchanges^17,19–22^, and empirical studies have focused on the relationship between pore parameters and water oxygen concentration^4,5,7,23,42^. Conversely, in these previous studies, the mechanical resistance of foraminiferal tests has never been considered, although it strongly influences the pore patterns, as shown by our model. To our knowledge, the only quantitative study on this topic was published by Wetmore^43^. After investigating various coastal taxa in different environmental settings, she concluded that test robustness increases with size and with increased physical stress (i.e. sediment coarseness, water turbulence). Our study confirms the major importance of resistance to mechanical constraints, and shows that the variability in pore patterns observed in *Ammonia* is not only an adaptation to increase gas exchanges (through increased overall porosity), but is at the same time strongly controlled by the mechanical resistance of the test.

### The foraminiferal dilemma: the choice between gas exchanges and test solidity

A higher porosity obtained by an increase in pore diameter accompanied by a reduction of pore density has been described for *Ammonia* sp. by Moodley & Hess^32^ and Petersen *et al*.^35^. The same strategy was also highlighted for planktonic foraminifera^44,45^, but has never been shown for other benthic foraminiferal taxa. Several authors have studied pore density, in relation to bottom water oxygen concentrations^4,5,7,23^. Unfortunately, in the latter studies, pore size was not investigated and the overall porosity was not taken into account. As highlighted in our study, the concomitant use of pore density and pore size is mandatory to understand foraminiferal pore patterns related to environmental conditions. In view of our results, studies that consider only one of these two parameters are potentially strongly biased. However, the study of total porosity, which represents a combination of pore number and pore size, should give an acceptable, albeit incomplete, description of pore patterns.

An alternative approach to increase porosity would be to increase both pore density and pore size. This strategy has been suggested for *Hanzawaia nitidula*, a foraminifer living in low oxygen bottom waters^24^, but unfortunately, no quantitative measurements have been presented to corroborate this interpretation. However, our scaling law model strongly suggests that such a strategy is not realistic, because simultaneously increasing pore size and pore density will rapidly lead to a strong decrease of the test robustness.

Our large empirical data set suggests that a higher porosity can only be attained without substantially decreasing test robustness by concurrently increasing the pore size and decreasing pore density. In *Ammonia* sp., a porosity of 30% is the upper threshold observed in our data. This corresponds to a pore density of about 10 pores per 562 µm² and a radius of about 2 µm for individual pore. All the individuals, irrespective of their age or geographical origin, plot on the same curve; this clearly indicates that the observed relationship between total porosity, pore density and pore radius reflects a strong control of metabolic and mechanicals constraints as predicted by our scaling law model.

The scaling law model presented here is based on the pore density and pore size in the last chamber. Due to the particular calcification process of foraminifera with a lamellar test, where with every newly formed chamber, a thin calcite layer is precipitated over the entire test, pore patterns will probably change with ontogeny. For instance, Petersen *et al*.^35^ showed that in *Ammonia*, toward earlier chambers, pore density was increasing, whereas pore size was decreasing, without a significant trend in overall porosity. However, they also noted that these ontogenetic changes were minimal compared to the differences observed between specimens from different sites. It should be noted as well that in spite of potential changes of pore patterns in successive chambers, each chamber was the last chamber when it was formed, and our model results were very probably valid at that moment.

An alternative way to adapt to low oxygen conditions would be to construct thinner tests. In fact, many calcareous taxa adapted to low oxygen environments are indeed characterised by very thin tests^34,37–39^. Just as increased porosity, also a thinner test would lead to a lower test robustness. The modelling of varying test thickness is the subject of ongoing research.

### Ecological considerations

In this study, the specimens included in the dataset belong to three different pseudocryptic (morphologically distinguished only after their identification by DNA analysis) phylotypes of the genus *Ammonia*: T1, T2 and T6^39,40^. As shown by Richirt *et al*.^41^, the phylotype T2 seems unable to attain a porosity higher than 20% and a mean pore radius larger than 0.7 µm (Figs 3–5, left cluster). T1 and T6 are able to attain higher overall porosity with upper limits of about 25% and 30% and a mean pore radius larger than 1.25 µm and 1.8 µm respectively (Figs 3–5, right cluster). Although these three phylotypes apparently respond to the same physical controls, as shown by the model (i.e. metabolic demand and mechanical resistance), they exhibit different values of mean pore radius, pore density and porosity, suggesting that they occupy different ecological niches. We hypothesize that these three phylotypes represent different adaptations to dissolved gas concentrations (i.e. oxygen concentration)^32,35^.

At equal porosity, individuals with large pores (phylotype T6) are more robust compared to individuals with small (phylotype T2) or intermediate pore sizes (phylotype T1). In view of the limited range of pore size (and total porosity) observed for each of the three phylotypes^25,40,41^, it appears that pore size is phylotype-dependant, as was already shown for other foraminiferal taxa^46,47^. However, within the limits observed for each of the three phylotypes, there is still substantial variability in porosity, which highlights a certain degree of ecophenotypic plasticity or intraspecific genetic variation. Intra-phylotype plasticity as an additional response to environmental conditions has earlier been shown in other protists^48^ (i.e. the number of pores in different phylotypes of testate amoeba). Disentangling genetic and environmental controlling factors is essential to better understand the observed morphological variability.

In order to improve gas exchanges and maintain mechanical integrity of the test, the optimal solution is to increase pore size while decreasing pore density. It appears that, compared to the two other phylotypes, T6 has developed a pore pattern maximising gas exchanges, while maintaining test robustness. This should allow T6 to better perform under low oxygen conditions, which are found immediately below the sediment surface in the intertidal and subtidal mudflats where these *Ammonia* phylotypes flourish. More detailed studies have to be carried out to investigate whether T6 is able to live deeper in the sediment (i.e. where the oxygen content is lower) than T1 and T2. It has been suggested that phylotype T6 has only recently been introduced in Europe, and originates from East Asia^49^. Its higher porosity and a potential increased tolerance to hypoxic conditions, could explain why the phylotype T6 has successfully colonized mudflats along the European coasts, including areas which were not occupied by T1 and T2 before its arrival, such as the Kiel Fjord^50^ or the Baltic Sea^51^.

### Proxy potential of foraminiferal pore patterns

The model developed in this study represents a first step to better understand foraminiferal pore patterns, and allowed the identification of the two main controlling factors in *Ammonia* sp. Although there is no reason to think that pore patterns in other biconvex foraminifera with a lamellar test do not follow the same systematics, the available data do not allow us to confirm this. Our study emphasizes the importance of examining both pore density and pore size when investigating foraminiferal pore patterns. Our results strongly suggest that in biconvex foraminifera with a lamellar test, different pore patterns are a morphological adaptation (genetically encoded and/or phenotypic) allowing the modulation of gas exchanges with the outer environment. Increased gas exchanges may be very beneficial in low oxygen environments. This observation has been the rationale behind the attempts to use pore patterns as a proxy for bottom water oxygen concentrations^4–7^. Our scaling law model is not restricted to exchanges of oxygen, but could also be applied to other dissolved compounds in water, such as nitrate. Recently, pore patterns in *Bolivina spissa* have been proposed as a proxy to reconstruct past nitrate concentrations^8,9^. Although the general test shape of *Bolivina* is very different compared to *Ammonia*, a theoretical scaling law model adapted to this genus, based on the same principles, should allow us to better understand how simultaneous changes of pore density and pore size will lead to maximal porosity, allowing optimisation of gas exchanges.

We are convinced that this type of modelling approach provides insight in the physical laws controlling foraminiferal porosity. Understanding the constraints controlling the foraminiferal porosity is a crucial prerequisite for the reliable calibration and successful application of paleoceanographic proxies based on foraminiferal pore patterns.

## Methods

### Scaling law model

Scaling arguments and dimensional analysis are extensively used to study the general characteristics of the biological world: the size and shape of plants and animals can be seen as nature’s adaptation to various constraints such as gravity, surface tension, viscosity, mechanical stress and so on. Under these constraints, most living organisms exhibit the notable property of self-similarity: they are prone to scaling laws and reproduce themselves as scales change. This study proposes scaling laws for foraminifera subjected to two constraints: the foraminifera need a minimal respiration rate in order to ensure a nominal metabolism (metabolic constraint) and the test mechanical resistance integrity (mechanical constraint). The model was developed for the last chamber, formed by a single calcite layer and which usually has the largest volume. Due to the thinner test wall, gas exchanges are more intense in this chamber, which is for the same reason also the most fragile.

#### Metabolic constraint

Considering the micrometric size of the pores, diffusion controls the transport of gas across the shell and Fick’s first law for steady-state diffusion of a gas through a porous material can be used:

$$\dot{m}={S}_{p}D\frac{{\rm{\Delta }}C}{L}$$

with $\dot{m}$ the flow-rate of gas, *D* the diffusion coefficient supposed to be constant and specific to the diffusive chemical species, *S*_*p*_ the pore area, ${\rm{\Delta }}C=x$ the concentration difference across the shell and *L* the test thickness. In order to keep a constant metabolic rate $\dot{m}=Cst$, and the equation above can be simplified to:

$$\frac{{S}_{p}x}{L}=Cst$$

The pore area depends on the number of pores *N* and on the pore radius *r* (the pore size distribution for a given test is roughly monodisperse)^35^:

$${S}_{p}=N\pi {r}^{2}$$

The metabolic constraint is thus:

$$\frac{N{r}^{2}x}{L}=Cst$$

We now assume that *N*, *r* and *L* are functions of *x* and scale like:

$$\begin{array}{ccc}N \sim {x}^{a} & r \sim {x}^{b} & L \sim {x}^{c}\end{array}$$

with *a*, *b* and *c* being constant to be determined. Re-writing the metabolic constraint, we obtain:

$${x}^{a+2b+1-c}=Cst$$

This condition must be true for any value of *x* implying:

$$a+2b+1-c=0$$

At this point we need other equations to solve the problem. They are provided by the mechanical constraint based on the resistance of the shell.

#### Mechanical constraint

We consider the test chamber as a spherical thin shell of radius *R*_*s*_ and thickness $L\ll {R}_{s}$. From the theory of linear elasticity, we know that the wall is subject to a uniform stress *σ*:

$$\sigma =\frac{{\rm{\Delta }}P\,{R}_{s}}{2L}$$

with Δ*P* the pressure difference across the test. The pressure jump is due to the osmotic pressure across the cell membrane and is related to the concentration difference Δ*C* = *x* by the law of Van’t Hoff:

$${\rm{\Delta }}P=RT\,{\rm{\Delta }}C=RTx$$

with *R* the ideal gas constant and *T* the thermodynamic temperature supposed to be constant. In bird eggs, the wall thickness *L* and the size of the chamber *R*_*s*_ are roughly proportional (i.e. ${\rm{L}} \sim {{\rm{R}}}_{{\rm{s}}}$)^52,53^ implying that the density of the egg is constant. Assuming a similar relation for the last chamber (which is the thinnest and most fragile chamber of the test) of foraminifera of the genus *Ammonia*, we hypothesize that the stress *σ* in the shell scales with the concentration difference across the shell *x* like:

$$\sigma  \sim x$$

The key point for the mechanical constraint is the way the test is likely to break: since the test is mainly composed of calcite, only very limited plastic deformation is possible, and in case of increasing mechanical stress, brittle fractures will rapidly occur, ultimately leading to breakage of the test. The failure theory predicts that the rupture occurs from a defect of characteristic size 𝑙 when the stress exceeds the limit σ* given by:

$${\sigma }^{\ast }=\frac{{K}_{c}}{\sqrt{\pi l}}$$

with *K*_*c*_ the stress intensity factor. We assume that the pores are such defects and that *l*~*r*. This implies that:

$${\sigma }^{\ast } \sim {r}^{-1/2} \sim {x}^{-b/2}$$

The test failure is reached when *σ* = σ* and the mechanical constraint implies that:

$$b=-\,2$$

Our last hypothesis is that *R*_*s*_ does not depend on *x*. Such an argument can be understood on simple geometrical grounds: the exchanges with the surrounding medium are proportional to the cell surface $S \sim {R}_{s}^{2}$ whereas its needs are proportional to its volume $V \sim {R}_{s}^{3}$. There must be a cell size above which the cell asphyxiates because the exchanges across the cell membrane are not fast enough. Therefore, the cell cannot grow indefinitely and reaches an optimal size *R*_*s*_. Because *R*_*s*_ ~ *L* ~ *x*^*c*^, the only acceptable solution is *c* = 0. Now the metabolic constraint gives:

$$a=-\,2b-1=3$$

From these values, the relationships between *N*, Φ, σ* and *r* can be obtained:

$$\begin{array}{ccc}N \sim {r}^{-3/2} & {\rm{\Phi }} \sim {r}^{1/2} & {\sigma }^{\ast } \sim {r}^{-1/2}\end{array}$$

### Mathematical optimisation

This approach consists in maximizing both porosity Φ (i.e. maximize transfer through the test) and mechanical resistance of the test (i.e. maximize σ*/*N*). If we suppose that pore density scales as *N* ~ *r*^*a*^ with *a* an exponent to be found, porosity scales as ${\rm{\Phi }} \sim N{r}^{2} \sim {r}^{a+2}$ (the limit stress always scales as ${\sigma }^{\ast } \sim {r}^{-1/2}$). Thus the best compromise is obtained by solving:

$$\frac{d}{dr}({\rm{\Phi }}+\frac{{\sigma }^{\ast }}{N})=\frac{d}{dr}({r}^{a+2}+\frac{1}{{r}^{\frac{1}{2}+a}})=0$$

After simplification, we obtain:

$${r}^{2a+5/2}=\frac{1+2a}{a+2}=Cst$$

The only solution to keep constant the left-hand side of the equation above is:

$$a=-\,5/4$$

The relationships between *N*, Φ, σ* and *r* finally write:

$$\begin{array}{ccc}N \sim {r}^{-5/4} & {\rm{\Phi }} \sim {r}^{3/4} & {\sigma }^{\ast } \sim {r}^{-1/2}\end{array}$$

### Empirical data

**Figure 6:**
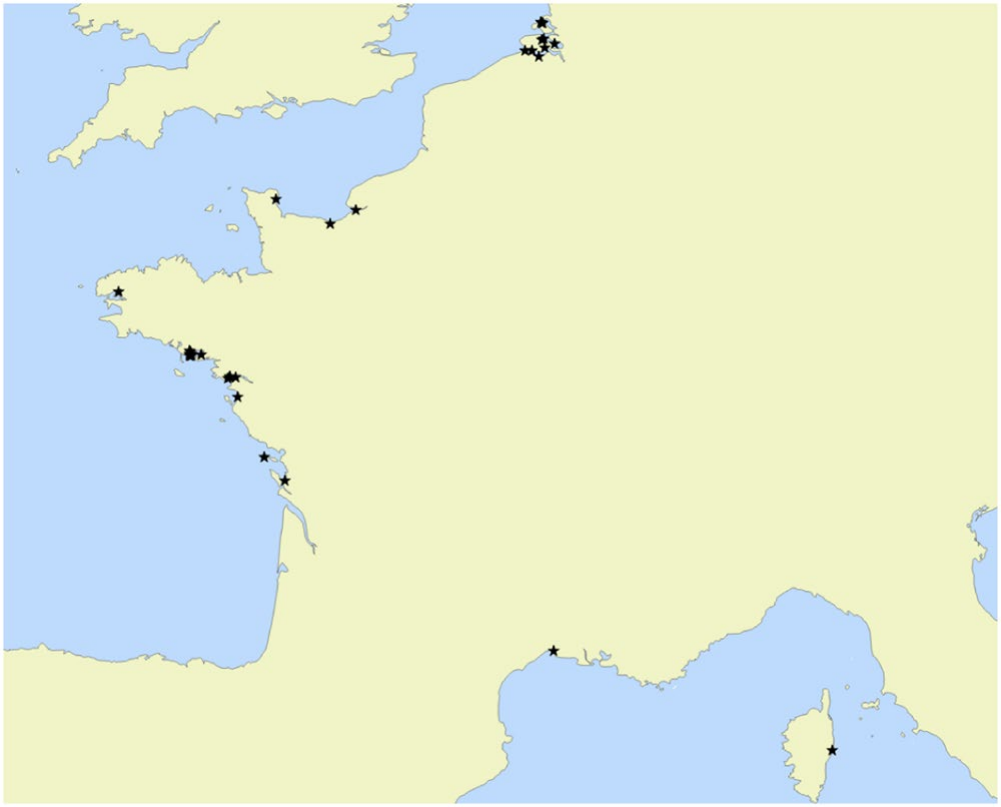
Map of the sampled stations.

The 1386 *Ammonia* sp. individuals were sampled at 36 different stations (see Fig. 6 and Table 1) around European coasts (see map), plus one station in Yokohama (Japan, three individuals) and one station at Tulear in Madagascar (one individual). Individuals come from fossil, recent and experimental material (only chambers formed in experimental conditions have been measured for specimens used in laboratory growth experiments), The measurements of the porosity features were performed following the methodology developed in Petersen *et al*.^35^. The measured range of pore diameter shows that the three phylotypes T1, T2 and T6 are mixed in the samples^41^. Pore pattern data generated or analysed during this study have been included in the supplementary information files.

**Table 1:** Location and number of individuals for each station (ordered by latitude – 1386 individuals in total).

Station	Latitude	Longitude	Number of individuals
Mokbaai, Texel	53°0′14.4″N	4°46′4.799″E	6
Grev-3	51°45′47.401″N	3°52′08.563″E	197
Grev-1	51°44′50.04″N	3°53′24.06″E	230
Grev-2	51°44.956 N	03°53.826E	193
Zandkreek	51°33′12.24″N	3°52′25.34″E	107
Escault 6	51°33.401′N	3°55.082′E	2
Escault 5	51°29.888′N	4°07.915′E	5
Biezelingse Ham	51°26′53.40″N	3°55′49.79″E	73
Escault 4	51°25.208′N	3°41.783′E	32
Escault 2	51°25.134′N	3°33.804′E	3
Escault 1	51°20.881′N	3°49.365′E	20
English Channel - Saint Vaast	49°34′38.60″N	1°16′38.80″W	4
English Channel - Estuaire de la Seine	49°26′31.30″N	0°16′25.20″E	13
English Channel - Ouistreham	49°16′16.40″N	0°14′12.20″W	3
Rade de Brest	48°24′13.10″N	4°21′16.00″W	12
Gulf of Morbihan - Bono	47°38′4.71″N	2°57′36.27″W	2
Fort Espagnol	47°36′47.50″N	2°57′11.10″W	8
Gulf of Morbihan - Toulvern	47°35′39.95″N	2°55′35.80″W	3
2C2	47°35′17.7″N	2°57′50.7″W	9
Auray River - KER2	47°34′60.00″N	2°57′17.20″W	40
Auray River - LOC1	47°34′12.12″N	2°56′32.40″W	15
Auray River - LOC2	47°34′11.58″N	2°56′26.58″W	50
Auray River - LOC3	47°34′11.29″N	2°56′21.38″W	6
Gulf of Morbihan - Bailleron est	47°34′38.07″N	2°44′45.25″W	4
St Pierre Lopérec	47°33′44.8″N	2°58′23.0″W	32
Loire - core SC05	47°17′10.30″N	2°10′31.80″W	30
Loire - Semhabel	47°17.293′N	2°10.906′W	16
Loire - RS2E	47°16′58.8″N	2°3′46.8″W	28
Saint Nazaire	47°15′56.75″N	2°13′20.79″W	69
Bourgneuf	47°0′56.38″N	2°1′31.00″W	124
Ile de Ré	46°13′23.13″N	01°30′46.27″W	2
Aiguillon	45°53′60.00″N	1°7′0.00″W	40
Gulf of Lion - Camargue	43°33′9.306″N	4°6′15.112″E	2
Corsica	42°8′7.44″N	09°31′59.04″E	2
Yokohama	35°19′21″N	139°38′6″E	3

## Supplementary information

Supplementary Information

## Data Availability

All data generated or analysed during this study are included in this published article (and its Supplementray Information files).
