# Supplementary material for: Scaling laws explain foraminiferal pore patterns
Source: Sci Rep. 2019 Jun 24;9:9149. doi: 10.1038/s41598-019-45617-x (PMC6591427; doi:10.1038/s41598-019-45617-x)
Supplement: Supplementary file 1 — Supplementary Information [file 41598_2019_45617_MOESM1_ESM.pdf]

Scaling laws explain foraminiferal pore patterns

Julien Richirt[1,*], Stéphane Champmartin[2,*], Magali Schweizer[1], Aurélia Mouret[1], Jassin Petersen[3], Abdelhak Ambari[2] and Frans J. Jorissen[1]

*Both co-authors contributed equally

[1] UMR 6112 LPG-BIAF Recent and Fossil Bio-Indicators, Angers University, 2 Bd Lavoisier, F-49045 Angers, France

[2] LAMPA, Arts et Métiers ParisTech, 2 Bd du Ronceray, BP 93525, 49035 Angers Cedex 01, France

[3] Institute of Geology and Mineralogy, University of Cologne, Zülpicher Str. 49a, 50674 Cologne, Germany

*Corresponding author: richirt.julien@gmail.com

Supplementary Information

Individual ID	Pore density (per 562 µm²)	Pore surface (%)	Pore radius (µm)
Au404	36.5	22.639	1.053
Au409	33.0	13.040	0.841
Au415	41.0	16.742	0.855
Au422	27.5	17.211	1.058
Au424	37.0	20.130	0.986
Au430	34.5	18.237	0.972
Au439	25.0	22.246	1.261
Au440	28.0	20.905	1.155
Au444	32.5	19.479	1.035
Au485	33.5	18.998	1.007
Au492	38.5	23.096	1.036
Au494	43.5	23.964	0.993
Au495	51.0	16.331	0.757
Au497	40.5	17.794	0.886
Au502	40.0	19.116	0.924
Yo060	49.5	18.762	0.823
Au398	42.0	20.096	0.925

Au402	44.0	19.721	0.895
Au403	39.5	22.122	1.001
Au406	30.5	22.906	1.159
Au407	39.5	16.050	0.852
Au408	40.5	20.607	0.954
Au411	46.0	13.082	0.713
Au419	39.0	25.692	1.085
Ma027	36.0	25.340	1.122
Ma142	28.0	26.807	1.308
Md013	32.5	21.940	1.099
Mo110	47.0	23.142	0.938
RB007	73.0	9.153	0.474
Re086	30.0	25.962	1.244
Re087	38.5	17.333	0.897
Yo051	40.0	22.709	1.008
Yo052	47.5	24.260	0.956
SN044	22.0	21.163	1.256
Au442	76.0	9.438	0.471
Au452	81.5	14.960	0.573
Au461	101.5	8.691	0.391
Au467	101.0	9.524	0.411
Au487	87.0	14.048	0.537
Au501	81.5	10.969	0.491
Au503	49.5	9.570	0.588
Co005	88.5	7.376	0.386
Co006	88.0	9.011	0.428
Ma030	106.5	12.524	0.459
Mo013	85.0	7.771	0.404
Mo017	93.5	7.889	0.388
Mo099	68.0	10.487	0.525
Mo101	92.0	10.871	0.460
Au400	86.0	13.613	0.532
Au423	101.0	17.822	0.562
Au451	96.0	13.943	0.510
Au453	91.5	13.677	0.517
Au462	86.0	13.709	0.534
Au491	80.0	15.544	0.589
Au500	81.5	14.433	0.563
Ma028	72.0	8.471	0.459
Ma031	74.0	8.238	0.446
Mo014	77.0	9.876	0.479
Mo098	57.5	14.104	0.662
Mo102	93.0	12.156	0.483
ZK020	32.5	24.662	1.165
ZK023	35.0	17.664	0.950
Ma150	95.5	12.131	0.477
Mo106	74.5	13.633	0.552
RB002	82.0	10.045	0.457
RB003	94.5	20.186	0.587
Ai052	29.0	27.249	1.296

Ai055	24.0	21.431	1.264
Ai056	19.0	21.878	1.435
Ai063	22.5	19.809	1.255
BH009	23.0	16.893	1.146
BH010	17.0	22.558	1.540
BH013	27.0	22.887	1.231
BH018	28.0	15.511	0.995
Bn097	22.0	17.980	1.209
Bn099	38.0	19.502	0.958
Bn108	22.5	18.353	1.208
Bn113	27.0	24.404	1.271
Bn116	39.5	21.047	0.976
Bn118	35.5	18.720	0.971
Bn119	35.0	30.271	1.244
Bn120	31.5	19.449	1.051
Li028	13.0	20.367	1.674
Li035	16.5	21.216	1.516
Ma080	27.0	33.440	1.488
Ma083	15.5	21.644	1.580
Ma084	20.5	29.109	1.593
Ma085	18.5	26.502	1.601
Ma086	23.0	19.678	1.237
Ma087	19.5	26.387	1.556
Ma089	13.0	22.987	1.778
Ma091	19.0	25.118	1.538
Ma094	16.5	23.947	1.611
Ma097	16.0	19.738	1.485
Ma101	22.5	19.749	1.253
Ma108	22.5	25.464	1.423
Ma109	26.0	19.138	1.147
Ma147	23.0	21.042	1.279
SN051	25.5	20.342	1.194
ZK043	25.5	25.384	1.273
ZK047	18.5	20.052	1.339
Ai045	24.0	17.034	1.025
Ai047	28.0	20.896	1.098
014.2 - Kerouarc'h	56.5	10.298	0.541
015.2 - Kerouarc'h	71.0	12.469	0.557
016.2 - Kerouarc'h	32.5	16.227	0.898
017.2 - Kerouarc'h	81.5	10.900	0.471
018.2 - Kerouarc'h	83.5	11.171	0.474
019.2 - Kerouarc'h	76.0	12.069	0.510
020.2 - Kerouarc'h	33.5	20.247	0.976
021.2 - Kerouarc'h	25.0	21.647	1.136
022.2 - Kerouarc'h	79.5	7.201	0.389
023.2 - Kerouarc'h	61.5	11.438	0.561
024.2 - Kerouarc'h	29.5	32.083	1.263
025.2 - Kerouarc'h	86.5	9.376	0.432
026.2 - Kerouarc'h	29.5	30.395	1.196
027.2 - Kerouarc'h (2)	33.0	27.364	1.183

028.2 - Kerouarc'h (2)	71.0	15.933	0.597
029.2 - Kerouarc'h	68.5	19.626	0.684
030.2 - Kerouarc'h	23.5	20.621	1.191
031.2 - Kerouarc'h (2)	115.0	4.148	0.251
032.2 - Kerouarc'h	103.5	9.846	0.404
033.2 - Kerouarc'h	126.0	5.329	0.274
034.2 - Kerouarc'h	50.0	10.407	0.582
035.2 - Kerouarc'h	31.0	15.955	0.916
036.2 - Kerouarc'h	51.5	10.010	0.581
037.3 - Kerouarc'h	30.5	23.213	1.059
038.2 - Kerouarc'h	86.5	13.189	0.509
039.2 - Kerouarc'h	71.0	10.528	0.491
040.2 - Kerouarc'h	24.5	18.775	1.095
041.2 - Kerouarc'h	25.0	21.343	1.110
042.2 - Kerouarc'h	71.5	13.781	0.555
043.2 - Kerouarc'h	35.0	23.731	1.005
044.2 - Kerouarc'h	90.5	12.830	0.494
045.2 - Kerouarc'h	71.5	9.620	0.470
054.2 - Locmariaquer	83.0	8.704	0.421
055.2 - Locmariaquer	92.0	7.570	0.370
056.2 - Locmariaquer	99.5	9.829	0.411
057.2 - Locmariaquer	84.0	13.173	0.512
058.2 - Locmariaquer	82.0	10.937	0.466
059.2 - Locmariaquer	77.5	10.298	0.471
060.2 - Locmariaquer	88.0	9.704	0.439
061.2 - Locmariaquer	61.0	14.932	0.622
062.2 - Locmariaquer	77.5	8.872	0.437
063.2 - Locmariaquer	50.0	13.458	0.662
065.2 - Locmariaquer	61.0	12.777	0.593
066.2 - Locmariaquer	77.5	12.806	0.513
067.2 - Locmariaquer	72.0	14.019	0.560
068.2 - Locmariaquer	92.5	15.080	0.514
069.2 - Locmariaquer	85.5	11.360	0.473
070.2 - Locmariaquer	80.0	9.647	0.451
071.1 - Locmariaquer	71.0	2.468	0.248
071.2 - Locmariaquer	78.5	4.031	0.293
072.2 - Locmariaquer	74.0	15.042	0.573
073.2 - Locmariaquer	110.5	15.224	0.487
074.2 - Locmariaquer	75.5	10.623	0.473
075.2 - Locmariaquer	95.5	15.228	0.522
076.2 - Locmariaquer	102.5	14.512	0.495
077.2 - Locmariaquer	87.5	14.790	0.530
078.2 - Locmariaquer	78.0	14.899	0.567
079.2 - Locmariaquer	66.5	11.365	0.528
080.3 - Locmariaquer	78.0	9.869	0.451
081.2 - Locmariaquer	60.5	9.538	0.516
082.2 - Locmariaquer	98.0	12.956	0.472
083.2 - Locmariaquer	87.0	8.104	0.408
084.2 - Locmariaquer	96.5	19.009	0.586
094.2 - St Pierre Loperec	77.5	13.190	0.546

095.2 - St Pierre Loperec	40.5	11.420	0.681
096.2 - St Pierre Loperec	59.5	14.568	0.643
097.2 - St Pierre Loperec	29.0	23.953	1.106
098.2 - St Pierre Loperec	70.0	12.848	0.539
100.2 - St Pierre Loperec	37.0	25.301	1.038
101.2 - St Pierre Loperec	72.0	14.994	0.583
102.2 - St Pierre Loperec	83.5	10.233	0.444
105.2 - St Pierre Loperec	38.5	20.141	0.905
107.2 - St Pierre Loperec	35.5	14.057	0.793
108.2 - St Pierre Loperec	50.5	7.513	0.494
109.2 - St Pierre Loperec	64.5	11.009	0.506
110.2 - St Pierre Loperec	71.0	13.610	0.574
111.2 - St Pierre Loperec	72.0	13.181	0.564
113.2 - St Pierre Loperec	28.5	18.097	1.006
114.2 - St Pierre Loperec	82.0	13.121	0.502
115.2 - St Pierre Loperec	76.5	14.934	0.548
116.2 - St Pierre Loperec	35.0	18.295	0.941
117.2 - St Pierre Loperec	44.5	24.113	0.929
119.2 - St Pierre Loperec	33.5	16.767	0.888
120.2 - St Pierre Loperec	96.0	12.997	0.471
121.2 - St Pierre Loperec	73.0	14.917	0.578
123.2 - St Pierre Loperec	74.0	16.584	0.605
124.2 - St Pierre Loperec	24.0	18.733	1.094
125.2 - St Pierre Loperec	88.5	17.289	0.565
126.2 - St Pierre Loperec	72.5	13.790	0.570
127.2 - St Pierre Loperec	39.0	10.871	0.681
128.2 - St Pierre Loperec	37.5	7.755	0.589
129.2 - St Pierre Loperec	100.0	19.333	0.566
130.2 - St Pierre Loperec	98.0	5.578	0.313
131.2 - St Pierre Loperec	70.5	8.423	0.451
132.2 - St Pierre Loperec	44.5	17.875	0.816
210.2 - Kerouarc'h	50.0	14.816	0.721
211.2 - Kerouarc'h	86.5	12.186	0.458
212.2 - Kerouarc'h	37.5	18.999	0.860
214.2 - Locmariaquer	77.0	9.361	0.460
215.2 - Locmariaquer	92.0	9.605	0.427
216.2 - Locmariaquer	75.0	9.072	0.456
217.2 - Locmariaquer	81.5	10.592	0.472
218.2 - Locmariaquer	54.0	7.374	0.465
219.2 - Locmariaquer	71.5	3.870	0.308
220.2 - Locmariaquer	87.5	8.701	0.412
221.2 - Locmariaquer	64.5	8.808	0.481
299.2 - Fort Espagnol	75.5	10.227	0.464
300.2 - Fort Espagnol	75.5	11.475	0.497
301.2 - Fort Espagnol	63.5	12.115	0.561
303.2 - Fort Espagnol	129.5	12.355	0.400
304.2 - Fort Espagnol	70.5	12.275	0.541
305.2 - Fort Espagnol	69.0	11.406	0.505
306.2 - Fort Espagnol	68.5	15.488	0.620
307.2 - Fort Espagnol	93.0	10.576	0.439

332.2 - 2C2-2016	25.5	26.539	1.258
333.2 - 2C2-2016	21.0	20.179	1.178
334.2 - 2C2-2016	111.5	10.353	0.398
336.2 - 2C2-2016	75.5	15.129	0.589
337.2 - 2C2-2016	60.0	13.457	0.609
338.2 - 2C2-2016	61.0	11.507	0.550
339.2 - 2C2-2016	29.0	25.637	1.179
340.2 - 2C2-2016	26.5	19.246	1.090
341.2 - 2C2-2016	19.0	26.614	1.439
ST1A	22.5	21.250	1.300
ST1B	22.0	20.820	1.301
ST1C	18.5	21.460	1.441
ST1D	22.0	28.690	1.527
ST1E	22.0	24.960	1.424
ST1F	12.5	15.750	1.501
ST1G	24.0	17.300	1.135
ST1H	40.0	20.970	0.969
ST1I	29.0	20.330	1.120
ST1I-DL	20.5	26.620	1.524
ST1J	20.0	21.040	1.372
ST2A	15.0	25.680	1.750
ST2B	20.5	17.370	1.231
ST2C	15.0	22.070	1.622
ST2D	25.0	27.310	1.398
ST2E	30.0	23.950	1.195
ST2F	26.0	18.160	1.117
ST2G	14.5	25.740	1.781
ST2H	12.5	24.790	1.883
ST2J	24.5	22.300	1.275
ST2K	11.5	28.660	2.111
ST3A	21.0	25.210	1.465
ST3B	29.5	24.550	1.219
ST3C	19.0	20.500	1.389
ST3E	22.5	26.640	1.455
ST3F	17.5	22.220	1.507
ST3H	15.5	13.340	1.241
ST3I	23.0	16.960	1.148
ST3J	23.0	24.490	1.380
ST3L	16.5	19.910	1.469
ST3M	20.0	19.780	1.329
1A	33.0	21.880	1.090
1B	32.0	17.940	1.001
1C	106.0	16.220	0.523
1D	18.5	24.760	1.547
1E	17.5	20.950	1.464
5B	134.5	13.260	0.418
5C	113.0	6.580	0.324
5D	41.5	23.950	1.016
10A	89.5	11.170	0.472
15A	91.5	13.680	0.517

20A	37.0	18.090	0.936
30A	59.0	5.370	0.403
30B	127.5	10.990	0.391
40C	30.0	21.470	1.131
40D	28.5	17.780	1.056
40E	18.5	19.340	1.367
40F	38.0	11.410	0.733
45B	56.0	15.720	0.709
45C	72.0	12.910	0.567
45D	26.5	22.500	1.232
45E	33.0	21.460	1.078
50A	42.5	19.770	0.911
50B	128.5	11.820	0.407
50C	37.0	20.240	0.989
50E	23.5	33.890	1.606
55A	25.5	18.680	1.145
55B	118.5	11.460	0.415
55D	87.0	11.180	0.479
55E	52.5	14.260	0.698
60B	21.0	8.430	0.846
60C	27.0	24.410	1.272
60D	100.5	12.350	0.469
60E	99.5	15.460	0.526
65A	111.0	10.100	0.403
65B	118.0	9.460	0.378
65C	24.5	20.490	1.223
65D	21.5	24.810	1.436
70A	21.0	16.600	1.189
70B	108.5	19.900	0.573
70C	86.5	12.480	0.508
75B	47.0	14.240	0.736
80C	33.5	22.230	1.090
80D	45.0	19.860	0.888
80E	25.5	24.410	1.309
80H	26.5	21.130	1.194
85A	116.5	10.170	0.395
85B	30.5	11.310	0.814
85D	38.5	22.910	1.031
85G	43.5	15.050	0.786
90A	16.0	18.110	1.423
90B	58.5	23.020	0.839
90C	133.0	11.290	0.391
90D	28.0	25.140	1.267
95A	24.5	16.700	1.104
95B	28.5	17.190	1.039
95C	29.0	23.260	1.198
95E	24.5	22.470	1.280
100A	50.0	19.330	0.831
100B	97.0	10.210	0.433
100C	120.5	9.000	0.366

100D	96.0	10.120	0.433
105A	91.5	10.380	0.451
105B	45.0	17.060	0.823
105C	102.5	6.360	0.334
105E	115.5	13.210	0.451
110A	106.0	12.840	0.465
110B	112.5	15.950	0.505
110E	148.0	6.260	0.276
110H	79.0	10.870	0.495
115A	83.0	7.030	0.391
120A	53.5	19.130	0.800
120B	52.0	9.920	0.584
120C	41.0	16.560	0.850
125A	85.0	11.720	0.495
125B	28.0	6.880	0.663
125D	21.5	23.090	1.385
125E	41.5	20.000	0.929
130B	42.5	19.970	0.917
130D	110.0	5.410	0.299
130E	144.5	6.330	0.282
130G	128.5	8.740	0.348
135B	92.0	12.940	0.501
135C	94.5	15.200	0.535
135D	103.0	8.030	0.374
135G	43.0	19.250	0.894
140A	142.0	11.200	0.374
140B	100.0	9.250	0.407
140C	109.5	14.650	0.489
145A	117.5	9.310	0.374
145C	81.0	20.520	0.672
145D	38.0	24.510	1.073
145E	119.5	8.200	0.352
150A	95.5	10.030	0.433
155A	67.0	10.370	0.526
155B	117.0	7.900	0.348
157A	42.5	21.310	0.947
157B	71.0	12.530	0.561
157C	92.5	15.070	0.541
157D-PA	56.0	9.460	0.550
158A	90.0	10.220	0.451
158B	155.0	14.650	0.411
158C	106.5	11.140	0.433
159A	80.0	12.380	0.526
159B	44.5	13.840	0.746
159C-PA	129.0	11.510	0.399
159D-PA	124.0	10.160	0.383
160C	88.0	12.370	0.501
160E	111.0	4.200	0.259
160F	48.5	12.870	0.689
160G	58.5	10.740	0.573

160I	126.5	10.670	0.387
161A	110.0	12.530	0.451
161B	44.0	9.900	0.633
161C	78.5	14.440	0.573
161D	104.5	12.180	0.455
162A	112.5	12.480	0.444
163A	86.0	7.810	0.403
163B	131.0	14.360	0.444
163C	37.5	7.710	0.608
163D-PA	92.0	7.520	0.383
164A	96.5	12.540	0.482
164B	109.0	9.000	0.383
164C	38.0	14.790	0.835
165A	97.0	9.450	0.418
165B	112.5	10.200	0.403
166A	115.5	9.780	0.387
166B	99.0	12.720	0.479
166C-PA	93.0	12.400	0.475
166D-PA	87.0	7.190	0.383
167A	104.0	12.240	0.458
168A	105.0	14.360	0.495
168B	89.5	12.760	0.505
168C	119.0	10.300	0.395
168D	33.0	19.340	1.023
169A	143.0	9.880	0.352
169B	124.0	9.410	0.370
169C	108.0	11.270	0.433
169D	52.5	5.190	0.426
170A	79.0	9.510	0.465
170B	106.0	11.350	0.437
170D	106.0	9.700	0.403
170E	88.0	11.950	0.492
175A	98.0	12.890	0.485
175B	95.0	16.000	0.550
175C	114.0	7.250	0.339
175D	98.5	6.820	0.352
180A	101.0	14.440	0.505
180B	122.5	10.860	0.399
180E	130.0	11.810	0.403
b1	27.0	20.780	1.173
b2	42.0	20.040	0.924
b3	19.5	27.030	1.575
b4	26.5	18.040	1.103
b5	31.0	17.080	0.992
b6	31.0	21.450	1.113
b7	32.0	20.680	1.075
b8	31.0	17.620	1.008
b9	20.5	20.700	1.343
b10	34.0	27.560	1.203
b11	29.0	21.190	1.144

z1	17.0	18.670	1.401
z2	25.5	23.590	1.287
z3	33.0	21.460	1.078
z4	18.0	16.430	1.278
z5	35.5	14.750	0.861
z6	19.5	22.380	1.433
z7	20.5	27.540	1.550
z8	16.0	18.920	1.454
z9	21.0	21.300	1.347
z10	23.5	27.170	1.438
z11	33.0	28.200	1.236
z12	19.0	17.220	1.273
a1	12.0	26.450	1.985
a2	25.5	15.960	1.059
a3	41.0	4.500	0.444
a4	38.5	20.020	0.964
a5	29.0	21.770	1.159
a6	35.0	16.970	0.930
a7	32.5	16.660	0.957
a8	20.5	18.860	1.283
a9	23.5	19.420	1.215
a10	28.5	16.290	1.011
a11	32.0	19.100	1.033
a12	21.5	21.960	1.352
a13	29.0	22.460	1.177
a14	22.0	19.470	1.258
r1	24.0	15.540	1.076
r2	23.5	14.740	1.059
r3	33.5	20.800	1.054
r4	28.5	17.700	1.054
r5	18.5	14.760	1.194
r6	28.0	16.880	1.039
r7	18.0	18.880	1.369
r8	47.0	19.950	0.870
r9	38.0	21.320	1.001
r10	28.0	29.350	1.369
r11	39.0	20.310	0.966
r12	27.5	25.840	1.296
r13	25.5	15.320	1.036
s1	27.5	16.190	1.026
s2	28.0	18.010	1.072
s3	17.5	28.860	1.718
s4	20.0	16.320	1.207
s5	18.0	17.670	1.326
s6	16.0	14.250	1.262
s7	20.5	17.230	1.226
s8	20.0	22.070	1.405
s9	19.0	20.240	1.380
s10	13.0	24.500	1.836
s11	24.0	20.770	1.244

s12	26.0	18.210	1.118
f1	24.5	10.430	0.872
f4	24.0	18.880	1.186
f7	42.5	18.780	0.888
m1	14.0	13.850	1.330
m2	25.0	18.400	1.147
m3	25.0	23.370	1.293
m4	24.5	16.430	1.095
m5	20.5	22.580	1.404
m6	30.5	18.480	1.040
img42	31.5	21.380	1.101
img44	36.5	25.330	1.114
img46	26.5	25.470	1.311
img48	26.5	26.140	1.328
img50	29.5	23.740	1.199
img52	29.0	26.450	1.277
img54	24.0	23.660	1.328
img56	48.5	22.070	0.903
img58	39.0	21.030	0.982
img60	29.0	25.790	1.262
img63	29.5	22.130	1.158
am1	47.0	29.190	1.054
am2-02	31.5	24.170	1.171
am2-03	36.5	16.870	0.910
am3-02	28.5	22.860	1.198
am3-03	33.5	21.040	1.060
h7-1	37.5	17.280	0.908
h7-2	35.5	18.410	0.962
h7-3	34.0	21.850	1.072
h7-4	41.0	20.850	0.954
h7-5	46.0	18.280	0.843
h14-1	51.0	16.280	0.755
h14-2	29.0	21.980	1.164
h14-3	35.5	17.200	0.930
h14-4	46.5	18.250	0.837
h14-5	41.0	19.800	0.929
h15-2	24.5	23.500	1.310
h15-4	40.0	15.360	0.829
h15-5	37.0	25.900	1.118
h15-6	40.5	25.510	1.062
h15-7	16.0	12.800	1.195
h17-1	24.0	16.250	1.100
h17-2	32.5	16.100	0.941
h17-3	37.0	15.730	0.872
h17-4	35.0	20.910	1.034
E1-02	92.5	6.910	0.366
E2-02	86.5	5.690	0.343
E3-02	78.5	11.250	0.505
E4-02	87.5	8.580	0.418
E5-02	80.5	4.960	0.334

E6-02	124.0	6.610	0.309
SEMA64a1-002	18.5	27.931	1.643
SEMA64a2-002	21.5	17.718	1.214
SEMA64a3-002	32.0	20.800	1.078
SEMA64a4-002	24.0	13.460	1.001
SEMA64a5-002	39.5	17.056	0.879
SEMA64a6-002	31.0	19.689	1.066
SEMA64a7-002	31.0	12.400	0.846
SEMA64a8-002	21.5	21.142	1.326
SEMA64a9-002	34.5	10.209	0.727
SEMA64a10-002	25.0	23.073	1.285
GREV1A23-002	20.0	19.920	1.335
GREV1A24-002	18.0	18.416	1.353
GREV2A17-002	23.0	25.509	1.408
GREV2A18-002	15.5	21.962	1.592
GREV2A19-002	27.0	25.436	1.298
GREV2A20-002	16.5	22.184	1.551
GREV2A21-002	18.0	23.291	1.521
GREV2A22-002	15.5	18.327	1.454
GREV3B11-002	13.0	18.624	1.601
GREV3B12-002	29.5	25.856	1.252
GREV3B13-002	17.5	16.864	1.313
GREV3B14-002	13.0	22.433	1.757
GREV3B15-002	21.5	19.653	1.279
GREV3B16-002	19.5	22.367	1.432
image65	20.0	13.853	1.113
image67	18.5	17.520	1.301
image69	20.0	16.213	1.204
image71	29.0	18.800	1.077
image73	25.0	17.182	1.109
image04	31.0	19.731	1.067
image05	18.0	12.509	1.115
image06	24.0	17.222	1.133
image08	26.0	20.276	1.181
image10	23.0	10.018	0.883
image12	23.0	22.484	1.322
image14	24.5	16.100	1.084
image16	30.5	16.633	0.988
image18	28.5	29.504	1.361
image20	23.5	14.169	1.038
image22	23.5	15.038	1.070
image24	24.0	19.640	1.210
image26	22.5	17.989	1.196
image28	31.0	12.564	0.851
image30	20.5	17.178	1.224
image32	21.0	16.180	1.174
image34	23.0	15.853	1.110
image36	21.0	14.820	1.123
image38	25.5	19.856	1.180
image40	23.0	17.147	1.155

image75	36.5	19.609	0.980
image77	31.0	15.464	0.944
image79	25.0	19.320	1.176
image83	26.0	21.131	1.206
image85	20.5	14.938	1.141
image87	22.5	19.369	1.241
image91	25.0	12.776	0.956
image93	17.5	16.873	1.313
image95	27.0	16.629	1.049
image97	20.0	21.918	1.400
image99	25.0	18.200	1.141
image101	34.5	14.993	0.882
image103	24.0	12.427	0.962
image105	23.5	21.476	1.278
image107	19.5	21.224	1.395
image109	23.5	13.084	0.998
image111	42.5	13.298	0.748
image113	22.0	19.991	1.275
image115	18.0	22.704	1.502
image117	33.0	20.487	1.054
image121	19.0	17.356	1.278
image123	29.0	21.271	1.145
image129	19.5	24.044	1.485
image133	32.5	23.276	1.132
image135	23.5	21.376	1.275
image137	18.5	20.364	1.403
image141	32.0	21.409	1.094
image145	25.0	23.589	1.299
image147	33.5	12.562	0.819
J1-02	70.5	8.320	0.458
J2-02	137.5	6.580	0.293
J3-02	97.0	4.650	0.309
J4-02	121.5	6.490	0.309
J5-02	79.0	2.840	0.252
J6-02	122.0	9.170	0.366
J7-02	60.0	8.170	0.495
J8-02	79.5	2.670	0.246
J9-02	112.0	6.910	0.334
J10-02	129.0	9.550	0.366
J11-02	108.0	4.840	0.282
J12-02	77.0	5.340	0.352
J13-02	71.0	4.240	0.329
J14-02	100.0	3.430	0.246
J16-02	59.0	2.390	0.271
J17-02	50.0	6.710	0.489
J18-02	48.5	2.940	0.329
J19-02	127.5	11.830	0.407
J21-02	117.5	13.790	0.458
J22-02	70.0	9.760	0.498
J23-02	74.0	5.340	0.361

J24-02	90.5	6.710	0.366
J25-02	81.5	7.920	0.418
J26-02	88.5	6.180	0.352
J27-02	51.0	5.130	0.422
J28-02	82.0	10.870	0.485
J30-02	113.5	8.950	0.374
J31-02	134.0	5.680	0.276
J32-02	99.0	12.880	0.482
J33-02	95.0	8.220	0.395
J34-02	47.0	9.090	0.589
J35-02	63.5	9.120	0.508
J36-02	94.0	6.430	0.348
J37-02	77.5	9.710	0.472
J38-02	84.0	9.200	0.444
J39-02	63.5	9.860	0.526
J40-02	87.0	6.350	0.361
AMMONIA-002	25.0	21.762	1.248
AMMONIA-004	28.5	23.540	1.215
AMMONIA-006	21.0	21.073	1.340
AMMONIA-008	16.0	16.424	1.355
AMMONIA-010	14.0	27.029	1.858
AMMONIA-012	23.5	23.358	1.333
AMMONIA-014	15.0	20.369	1.558
AMMONIA-016	18.5	16.931	1.279
AMMONIA-018	15.0	21.129	1.587
AMMONIA-020	18.0	19.609	1.396
AMMONIA-022	12.5	26.342	1.941
AMMONIA-024	18.5	21.349	1.437
AMMONIA-026	12.5	22.602	1.798
AMMONIA-028	18.0	22.049	1.480
AMMONIA-030	18.5	25.300	1.564
AMMONIA-032	14.5	23.558	1.704
AMMONIA-034	21.5	17.371	1.202
AMMONIA-036	19.0	20.922	1.403
AMMONIA-038	11.0	22.840	1.927
AMMONIA-040	29.0	15.458	0.976
AMMONIA-042	14.0	27.707	1.881
AMMONIA-044	21.0	22.222	1.376
AMMONIA-046	17.5	13.593	1.179
AMMONIA-048	15.5	21.629	1.580
AMMONIA-052	23.0	18.940	1.213
AMMONIA-054	20.0	22.929	1.432
AMMONIA-056	20.5	25.056	1.478
AMMONIA-058	20.5	17.731	1.244
AMMONIA-060	21.0	19.767	1.297
AMMONIA-062	23.0	22.504	1.323
AMMONIA-064	25.5	17.727	1.115
AMMONIA-066	22.0	13.851	1.061
AMMONIA-068	21.0	22.718	1.391
AMMONIA-070	28.5	16.887	1.029

AMMONIA-072	20.0	32.367	1.701
AMMONIA-074	15.5	27.093	1.768
AMMONIA-076	21.5	25.098	1.445
AMMONIA-078	25.0	23.898	1.307
AMMONIA-080	21.5	11.469	0.977
AMMONIA-082	18.0	30.838	1.750
AMMONIA-084	23.5	26.120	1.410
AMMONIA-086	26.0	20.720	1.194
AMMONIA-088	35.0	19.718	1.004
AMMONIA-090	24.5	24.891	1.348
AMMONIA-092	10.0	27.158	2.204
AMMONIA-094	21.0	29.411	1.583
AMMONIA-096	11.0	26.604	2.080
AMMONIA-098	26.0	24.284	1.292
AMMONIA-100	21.5	25.002	1.442
AMMONIA-102	15.5	26.749	1.757
AMMONIA-104	24.5	31.851	1.525
AMMONIA-106	23.0	19.244	1.223
AMMONIA-108	21.0	25.169	1.464
AMMONIA-110	20.5	18.798	1.280
AMMONIA-112	23.0	26.924	1.447
AMMONIA-114	12.5	23.558	1.836
AMMONIA-116	15.0	21.864	1.615
AMMONIA-118	21.0	30.322	1.607
AMMONIA1-002	16.5	13.493	1.209
AMMONIA1-004	31.0	19.649	1.065
AMMONIA1-006	18.0	17.362	1.313
AMMONIA1-008	28.0	17.198	1.048
AMMONIA1-010	14.5	15.718	1.392
AMMONIA1-012	15.5	13.704	1.257
AMMONIA1-014	10.0	11.749	1.449
AMMONIA1-016	11.5	21.204	1.816
AMMONIA1-018	17.5	20.216	1.437
AMMONIA1-020	14.5	16.504	1.427
AMMONIA1-022	21.0	17.522	1.221
AMMONIA1-024	20.0	18.611	1.290
AMMONIA1-026	18.0	19.787	1.402
AMMONIA1-028	8.0	11.376	1.595
AMMONIA1-030	15.0	17.842	1.458
AMMONIA1-032	15.0	16.933	1.421
AMMONIA1-034	15.5	22.416	1.608
AMMONIA1-036	14.0	18.687	1.545
AMMONIA1-038	24.0	11.049	0.907
AMMONIA1-040	12.5	19.842	1.685
AMMONIA1-043	16.0	21.033	1.533
AMMONIA1-045	13.0	11.851	1.277
AMMONIA1-047	14.0	20.591	1.622
AMMONIA1-049	24.5	19.056	1.179
AMMONIA1-051	15.0	18.873	1.500
AMMONIA1-053	13.5	13.356	1.330

AMMONIA1-055	15.5	22.136	1.598
AMMONIA1-057	14.0	15.869	1.424
AMMONIA1-059	21.5	22.258	1.361
AMMONIA1-061	16.0	23.580	1.623
AMMONIA1-063	19.5	20.484	1.371
AMMONIA1-065	15.5	21.242	1.565
AMMONIA1-067	14.0	14.453	1.359
AMMONIA1-070	13.5	17.120	1.506
AMMONIA1-072	17.0	23.156	1.561
AMMONIA1-074	11.5	10.676	1.288
AMMONIA1-076	10.0	16.540	1.720
AMMONIA1-078	17.5	18.922	1.390
AMMONIA1-080	12.5	15.131	1.471
AMMONIA1-082	27.5	18.324	1.092
AMMONIA1-084	18.5	17.598	1.304
AMMONIA1-086	16.5	18.216	1.405
AMMONIA1-088	18.0	12.644	1.121
AMMONIA1-090	19.5	19.567	1.340
AMMONIA1-092	17.5	15.331	1.252
AMMONIA1-094	13.5	20.067	1.630
AMMONIA1-096	21.5	14.676	1.105
AMMONIA1-098	16.5	18.153	1.403
AMMONIA1-100	17.5	13.878	1.191
AMMONIA1-102	13.0	18.484	1.595
AMMONIA1-104	16.5	20.642	1.496
AMMONIA1-106	24.0	17.704	1.149
AMMONIA1-108	19.0	10.258	0.983
AMMONIA1-110	12.0	20.367	1.742
AMMONIA1-112	20.0	15.440	1.175
AMMONIA1-114	17.0	18.107	1.380
AMMONIA1-116	14.0	17.262	1.485
AMMONIA1-118	15.5	23.411	1.643
AMMONIA1-120	22.5	15.202	1.099
AMMONIA1-122	15.0	16.751	1.413
E1-002	12.5	21.273	1.745
E2-002	15.5	25.329	1.709
E3-002	17.0	16.771	1.328
E4-002	18.0	21.871	1.474
E5-002	19.5	10.800	0.995
E6-002	7.5	17.693	2.054
E7-002	22.0	22.367	1.348
E8-002	19.5	26.040	1.545
E9-002	25.0	19.262	1.174
E10-002	17.5	17.898	1.352
E11-002	19.0	21.591	1.426
E12-002	15.0	18.638	1.491
E13-002	8.0	18.896	2.055
E14-002	15.5	23.293	1.639
E15-002	24.0	22.753	1.302
E16-002	17.0	17.798	1.368

E17-002	12.0	25.933	1.966
E18-002	14.5	16.156	1.411
E19-002	17.5	22.131	1.504
E20-002	17.5	23.696	1.556
E21-002	12.5	29.744	2.063
E22-002	16.5	25.644	1.667
E23-002	21.0	26.344	1.498
E24-002	12.5	20.602	1.717
E25-002	19.0	17.142	1.270
AMMONIA1-124	27.0	12.396	0.906
AMMONIA1-126	22.5	14.722	1.082
AMMONIA1-129	19.5	14.040	1.135
AMMONIA-131	18.0	17.404	1.315
AMMONIA1-133	21.5	10.924	0.953
AMMONIA1-135	14.5	8.929	1.049
AMMONIA1-137	14.5	18.433	1.508
AMMONIA1-139	19.0	15.673	1.215
AMMONIA1-141	17.5	19.882	1.425
AMMONIA1-143	22.0	17.531	1.194
AMMONIA1-145	12.0	19.978	1.725
AMMONIA1-147	13.0	13.876	1.382
AMMONIA1-149	25.5	22.602	1.259
AMMONIA1-151	14.5	19.198	1.539
AMMONIA1-153	19.5	19.027	1.321
AMMONIA1-155	16.5	19.631	1.459
AMMONIA1-157	14.0	16.640	1.458
AMMONIA1-159	7.5	16.189	1.965
AMMONIA1-161	19.0	16.847	1.259
AMMONIA1-163	12.0	17.936	1.635
AMMONIA1-165	20.0	16.793	1.225
AMMONIA1-167	10.0	12.411	1.490
AMMONIA1-169	19.0	20.902	1.403
AMMONIA1-171	16.0	21.651	1.556
AMMONIA1-173	7.5	20.260	2.198
AMMONIA1-175	11.5	15.764	1.566
AMMONIA1-177	11.5	16.893	1.621
AMMONIA1-178A	22.5	6.896	0.740
AMMONIA1-180	14.5	19.360	1.545
AMMONIA1-182	28.0	18.424	1.085
A122	23.0	20.462	1.261
A124	28.5	27.260	1.308
A126	26.0	22.956	1.257
A128	27.0	13.082	0.931
A130	18.5	18.113	1.323
A132	13.5	20.091	1.631
A134	24.0	21.120	1.254
A136	18.0	24.669	1.565
A138	15.5	17.542	1.423
A140	24.0	18.618	1.178
A142	15.5	19.456	1.498

A144	19.5	16.664	1.236
A146	16.0	14.882	1.290
A148	17.0	17.467	1.355
A150	12.0	23.556	1.874
A152	18.5	17.184	1.289
A154	20.0	21.084	1.373
A156	18.0	21.711	1.469
A158	21.5	14.522	1.099
A160	15.5	20.191	1.526
A162	23.5	17.029	1.138
A164	22.0	17.218	1.183
A166	18.0	17.393	1.314
A168	11.5	19.122	1.724
A170	18.0	19.713	1.399
A172	18.0	25.147	1.581
A174	22.5	20.522	1.277
A176	23.5	17.429	1.152
A178	14.5	20.282	1.582
A180	11.5	19.584	1.745
A182	18.0	18.793	1.366
A184	21.0	20.822	1.332
A186	16.0	19.796	1.487
A188	16.0	22.264	1.577
A190	17.5	16.040	1.280
A192	19.0	13.947	1.146
A194	22.0	16.858	1.171
A196	22.5	21.956	1.321
A198	15.5	18.242	1.451
A200	14.0	15.967	1.428
A202	14.0	22.587	1.698
A204	21.0	15.651	1.154
A206	18.5	14.607	1.188
A208	16.0	19.169	1.464
A210	13.5	18.704	1.574
A212	22.0	19.542	1.260
A214	21.0	18.689	1.262
A216	22.5	14.827	1.086
A218	17.0	18.878	1.409
A220	17.0	21.722	1.512
A222	18.0	18.809	1.367
A224	15.5	19.769	1.510
A226	12.0	19.540	1.706
A228	20.5	21.293	1.363
A230	14.5	19.207	1.539
A232	20.0	18.147	1.274
A234	16.5	19.187	1.442
A236	23.0	27.936	1.474
A238	31.5	20.936	1.090
A240	28.0	18.116	1.076
A242	15.0	18.564	1.488

A244	18.5	23.764	1.516
A246	12.0	20.022	1.727
A248	15.5	20.996	1.556
A250	21.0	18.996	1.272
A252	16.0	20.222	1.503
A254	15.0	12.920	1.241
A256	21.0	17.678	1.227
A258	12.5	17.569	1.585
A260	13.5	18.524	1.566
A262	19.0	19.602	1.358
A264	20.5	16.153	1.187
A266	15.0	21.753	1.610
A268	14.5	14.671	1.345
A270	17.5	17.898	1.352
A272	18.5	16.438	1.261
A274	20.0	22.709	1.425
A276	14.5	22.438	1.663
A279	22.0	12.718	1.017
A281	14.0	14.062	1.340
A283	15.0	20.660	1.569
A285	18.5	22.507	1.475
A287	19.5	14.198	1.141
A289	16.0	20.471	1.513
A291	12.5	12.811	1.354
A293	16.0	15.358	1.310
A295	18.5	16.636	1.268
A297	24.5	21.824	1.262
A299	17.0	15.213	1.265
A301	15.0	20.824	1.576
A303	19.5	15.638	1.197
A305	19.5	21.056	1.390
A307	14.0	23.238	1.723
A309	21.0	14.927	1.127
A311	18.0	14.453	1.198
A313	10.0	22.596	2.010
A315	20.5	16.736	1.208
A317	21.0	13.511	1.073
A319	18.5	20.689	1.414
A321	22.5	15.664	1.116
A323	16.5	15.884	1.312
A325	24.0	21.236	1.258
A327	16.5	13.609	1.214
A329	13.5	15.460	1.431
A331	18.5	22.900	1.488
A333	16.0	20.251	1.504
A335	16.5	18.760	1.426
A337	18.5	21.922	1.456
A339	11.5	15.564	1.556
A341	18.5	23.202	1.498
A343	26.5	23.513	1.260

A346	6.5	19.740	2.330
A348	20.5	14.598	1.128
A350	20.0	17.424	1.248
A352	22.5	16.473	1.144
A354	16.0	21.807	1.561
A356	21.0	30.978	1.624
A358	29.0	20.307	1.119
A360	18.0	13.553	1.160
A362	15.5	21.724	1.583
A002	16.5	20.138	1.477
A004	10.5	16.033	1.652
A006	19.0	12.769	1.096
A008	24.5	22.722	1.288
A010	14.5	20.818	1.602
A012	17.0	16.476	1.316
A014	15.0	8.909	1.031
A016	23.5	17.167	1.143
A018	19.5	15.280	1.184
A020	23.5	18.782	1.195
A022	26.0	24.220	1.291
A024	25.5	13.460	0.971
A026	16.0	21.560	1.552
A028	16.5	18.156	1.403
A030	18.5	22.078	1.461
A032	15.5	18.827	1.474
A034	14.5	18.236	1.500
A036	20.0	25.373	1.506
A038	28.5	10.229	0.801
A040	21.0	17.989	1.238
A042	13.5	24.609	1.805
A044	16.5	15.438	1.293
A046	17.0	17.740	1.366
A048	20.5	17.569	1.238
A050	26.5	19.153	1.137
A052	28.5	13.044	0.905
A054	27.0	17.400	1.073
A056	28.5	18.053	1.064
A058	10.0	15.271	1.653
A060	21.0	18.056	1.240
A062	22.0	23.609	1.385
A064	20.5	19.353	1.299
A066	26.5	23.393	1.256
A068	16.5	18.587	1.419
A070	19.5	20.002	1.354
A072	28.5	12.558	0.888
A74	16.5	21.460	1.525
A076	13.5	19.889	1.623
A078	26.5	20.058	1.163
A080	12.5	19.207	1.658
A082	19.5	19.684	1.344

A084	15.5	17.300	1.413
A086	20.0	18.858	1.298
A088	19.0	9.093	0.925
A090	21.0	18.487	1.255
A092	15.5	19.898	1.515
A094	17.5	26.400	1.642
A096	17.0	17.071	1.340
A098	25.0	12.882	0.960
A100	23.5	18.184	1.176
A102	25.0	17.391	1.115
A104	14.5	17.544	1.471
A106	19.5	22.247	1.428
A108	24.0	11.158	0.912
A110	17.0	24.013	1.589
A112	16.5	16.153	1.323
A114	15.5	17.104	1.405
A116	17.5	15.524	1.260
A118	30.0	23.947	1.195
A120	16.0	15.698	1.325
G1-002	86.5	13.982	0.538
G2-002	28.5	31.296	1.401
G3-003	18.0	19.100	1.377
G4-003	21.0	25.649	1.478
G5-002	24.0	18.813	1.184
G6-002	18.5	21.960	1.457
G7-002	19.5	20.429	1.369
G8-002	21.0	18.393	1.251
G9-002	26.5	20.149	1.166
G10-003	24.0	21.569	1.268
G11-002	28.0	21.907	1.183
G12-002	23.5	16.656	1.126
G13-002	34.0	20.749	1.045
G14-002	17.0	25.953	1.652
G15-003	19.0	22.373	1.451
G16-003	19.0	21.000	1.406
G17-003	19.5	20.951	1.386
G18-002	14.0	24.547	1.771
G19-003	16.0	15.858	1.331
G20-003	27.0	23.042	1.235
G21-002	31.5	25.936	1.213
G22-003	27.0	23.676	1.252
G23-002	32.0	25.053	1.183
G24-003	22.5	23.360	1.363
G25-002	18.5	28.653	1.664
G26-002	18.0	28.029	1.669
G27-003	25.5	20.931	1.212
G28-002	19.0	24.153	1.508
G29-002	41.5	23.193	1.000
G30-002	25.5	17.909	1.121
G31-002	27.0	21.860	1.203

ZK1	16.0	24.153	1.643
ZK2	27.5	15.804	1.014
ZK3	28.5	15.204	0.977
ZK4	20.5	27.049	1.536
ZK5	25.0	20.869	1.222
ZK6	20.0	21.913	1.400
ZK7	21.0	24.042	1.431
ZK8	18.5	27.942	1.643
ZK9	20.5	19.467	1.303
ZK10	28.5	29.109	1.351
ZK11	28.5	21.709	1.167
ZK12	16.5	19.036	1.436
ZK13	30.0	22.089	1.147
ZK14	23.0	21.389	1.290
ZK15	19.0	24.216	1.510
ZK16	26.0	14.191	0.988
ZK17	24.0	20.444	1.234
ZK18	21.0	19.433	1.286
ZK19	23.0	21.407	1.290
ZK20	19.5	20.538	1.372
ZK21	74.0	12.944	0.559
ZK22	16.0	22.167	1.574
ZK23	68.0	14.256	0.612
ZK24	66.5	19.360	0.722
ZK25	24.5	17.447	1.128
ZK26	14.5	23.420	1.699
ZK27	85.5	10.160	0.461
ZK28	28.0	28.616	1.352
ZK29	18.0	24.524	1.561
ZK30	23.5	20.240	1.241
ZK31	10.0	21.729	1.971
ZK32	10.0	19.591	1.872
ZK33	36.0	24.493	1.103
ZK34	30.0	16.102	0.980
ZK35	48.0	9.573	0.597
ZK36	38.5	16.460	0.874
ZK37	15.5	21.307	1.568
ZK38	75.5	13.047	0.556
ZK39	79.5	14.978	0.580
ZK40	101.0	13.529	0.489
ZK41	23.0	20.887	1.274
ZK42	15.5	20.238	1.528
ZK43	92.0	12.024	0.483
ZK44	14.0	19.182	1.565
ZK45	23.0	20.609	1.266
ZK46	20.0	19.909	1.334
ZK47	22.0	14.804	1.097
ZK48	16.0	26.447	1.719
ZK49	29.0	20.018	1.111
ZK50	15.0	20.591	1.567

ZK51	33.5	17.853	0.976
ZK52	16.5	24.129	1.617
ZK53	14.5	19.569	1.553
ZK54	21.0	19.282	1.281
ZK55	21.0	18.538	1.256
ZK56	22.0	27.476	1.494
ZK57	20.0	14.638	1.144
ZK58	20.5	21.409	1.367
ZK59	19.5	19.762	1.346
ZK60	13.0	17.120	1.535
ZK61	34.0	24.807	1.142
ZK62	89.0	13.436	0.520
ZK63	53.0	14.109	0.690
ZK64	39.0	25.649	1.084
ZK65	34.0	18.889	0.997
ZK66	55.0	19.322	0.793
ZK67	85.0	6.207	0.361
ZK68	72.5	11.800	0.539
ZK69	64.5	12.442	0.587
ZK70	34.5	20.660	1.035
ZK71	27.5	25.071	1.277
ZK72	71.5	12.902	0.568
ZK73	46.5	10.464	0.634
ZK74	45.0	9.702	0.621
ZK75	25.5	24.598	1.313
ZK76	16.0	16.693	1.366
ZK77	99.0	16.098	0.539
ZK78	58.0	5.371	0.407
ZK79	66.0	11.531	0.559
ZK80	78.0	11.284	0.509
SN54	33.5	14.642	0.884
SN55	25.5	19.782	1.178
SN56	29.0	12.511	0.878
SN57	28.5	12.767	0.895
SN58	24.5	18.271	1.155
SN59	28.0	18.009	1.072
SN60	13.0	22.447	1.757
SN61	26.0	16.922	1.079
SN62	22.5	15.402	1.106
SN63	28.5	14.718	0.961
SN64	23.0	20.736	1.270
SN65	30.0	13.800	0.907
SN66	24.0	16.371	1.104
SN67	19.0	20.558	1.391
SN68	18.5	15.247	1.214
SN69	31.5	25.789	1.210
SN70	20.0	19.560	1.322
SN71	18.0	21.193	1.451
SN72	29.5	20.209	1.107
SN73	28.0	21.649	1.176

SN74	24.5	17.227	1.121
SN75	23.5	15.320	1.080
SN76	27.0	20.576	1.167
SN77	21.0	18.271	1.247
SN78	27.5	20.711	1.160
SN79	20.0	21.849	1.398
SN80	17.5	22.909	1.530
SN81	22.0	18.669	1.232
SN82	29.0	16.169	0.999
SN83	23.0	18.816	1.209
SN84	29.0	13.429	0.910
SN85	23.0	11.451	0.944
SN86	30.5	23.893	1.184
SN87	27.5	15.167	0.993
SN88	35.5	17.418	0.937
SN89	25.5	17.933	1.121
SN90	21.0	18.469	1.254
SN91	30.5	22.471	1.148
SN92	23.0	20.584	1.265
SN93	27.0	21.969	1.206
SN94	11.0	22.371	1.907
SN95	13.5	13.791	1.352
SN96	16.0	19.133	1.462
SN97	15.5	17.893	1.437
SN98	15.5	25.069	1.701
SN100	26.5	21.362	1.201
SN101	28.0	20.898	1.155
SN102	28.5	18.296	1.071
SN103	19.0	22.722	1.462
SN104	17.0	34.716	1.911
SN105	30.5	20.196	1.088
SN106	31.5	17.542	0.998
SN107	37.0	16.551	0.894
SN108	24.5	20.698	1.229
SN110	21.5	12.876	1.035
SN111	24.0	19.342	1.200
SN112	13.0	22.782	1.770
SN113	18.5	17.460	1.299
SN114	18.0	15.162	1.227
SN115	26.0	17.564	1.099
SN116	25.0	19.031	1.167
SN118	22.5	18.769	1.221
SN119	22.5	15.082	1.095
SN120	19.0	18.349	1.314
SN121	23.0	17.342	1.161
SN122	17.0	19.407	1.429
SN123	26.0	12.711	0.935
RD1	90.0	13.011	0.508
RD2	81.0	13.264	0.541
RD3	95.5	20.324	0.617

RD4	76.0	10.313	0.493
RD5	90.0	16.051	0.565
RD6	60.0	8.229	0.495
RD7	35.5	27.273	1.172
RD8	68.0	10.362	0.522
RD9	55.0	8.002	0.510
E1	17.0	23.898	1.585
E1-2	27.0	17.513	1.077
E1-3	30.5	18.007	1.027
E1-4	29.0	17.351	1.034
E1-5	29.5	20.184	1.106
E1-6	30.0	16.073	0.979
E1-7	26.5	19.942	1.160
E1-8	39.0	20.567	0.971
E1-9	29.0	17.360	1.035
E1-10	39.0	16.173	0.861
E1-11	30.0	18.782	1.058
E1-12	37.0	22.993	1.054
E1-13	40.0	16.629	0.862
E1-14	25.0	17.051	1.104
E1-15	27.5	20.756	1.162
E1-16	27.5	14.736	0.979
E1-17	34.0	17.751	0.966
E1-18	44.5	14.504	0.763
E1-19	44.0	9.864	0.633
E1-20	57.0	13.667	0.655
E2	27.0	16.733	1.053
E2-2	14.5	21.302	1.621
E2-3	21.5	16.538	1.173
E4	22.5	19.680	1.251
E4-2	22.5	15.416	1.107
E4-3	22.5	21.878	1.319
E4-4	30.0	13.589	0.900
E4-5	27.5	19.358	1.122
E4-6	20.5	17.467	1.234
E4-7	29.5	16.493	1.000
E4-8	24.0	16.900	1.122
E4-9	27.0	18.658	1.112
E4-10	25.0	18.262	1.143
E4-11	31.0	13.404	0.879
E4-12	22.5	22.440	1.335
E4-13	35.0	15.342	0.885
E4-14	22.0	19.078	1.245
E4-15	19.5	19.229	1.328
E4-16	24.0	15.809	1.085
E4-17	22.0	19.587	1.262
E4-18	15.5	16.738	1.450
E4-19	30.0	14.973	0.945
E4-20	25.5	17.831	1.118
E4-21	25.5	12.716	0.944

E4-22	31.0	15.580	0.948
E4-23	30.0	19.060	1.066
E4-24	20.5	22.147	1.390
E4-25	20.0	16.838	1.227
E4-26	31.0	17.693	1.010
E4-27	37.5	13.358	0.798
E4-28	34.0	15.364	0.899
E4-29	29.0	19.549	1.098
E4-30	24.5	19.220	1.184
E4-31	22.0	23.947	1.395
E4-32	35.0	11.638	0.771
E5-1	60.0	7.333	0.468
E5-2	73.0	7.956	0.441
E5-3	69.0	7.109	0.429
E5-4	68.5	7.971	0.456
E5-5	30.0	4.193	0.500
E6-2	62.5	6.811	0.441
E6-3	80.0	13.320	0.546
BH	36.0	17.516	0.933
BH2	25.0	28.018	1.416
BH3	27.0	18.562	1.109
BH4	13.5	13.536	1.339
BH5	25.0	17.551	1.120
BH6	34.0	19.996	1.025
BH7	47.0	12.536	0.691
BH8	21.5	20.571	1.308
BH9	27.5	24.729	1.268
BH10	24.5	16.629	1.102
BH11	28.5	18.360	1.073
BH12	33.5	21.991	1.083
BH13	32.5	17.056	0.969
BH14	26.5	22.069	1.220
BH15	42.0	18.424	0.886
BH16	20.0	13.569	1.101
BH17	31.0	18.124	1.022
BH18	26.0	21.062	1.204
BH19	20.5	21.347	1.365
BH20	32.5	22.220	1.106
BH21	26.0	14.216	0.989
BH22	30.0	12.136	0.851
BH23	25.5	19.062	1.156
BH24	28.5	16.580	1.020
BH25	28.5	18.233	1.070
BH26	33.5	18.024	0.981
BH27	26.0	21.062	1.204
BH28	29.5	13.771	0.914
BH29	39.0	23.053	1.028
BH30	37.5	17.207	0.906
BH31	27.0	15.313	1.007
BH32	23.5	20.531	1.250

BH33	26.5	18.256	1.110
BH34	30.5	17.791	1.021
BH35	21.0	15.547	1.151
BH36	26.5	22.438	1.230
BH37	32.5	20.600	1.065
BH38	33.0	18.704	1.007
BH39	26.5	15.338	1.017
BH40	46.5	18.336	0.857
L1	78.5	14.344	0.572
L5	79.5	10.776	0.492
L6	79.0	8.642	0.442
L7	93.0	10.224	0.443
L8	73.0	7.749	0.436
L10	73.0	8.298	0.451
L11	98.0	9.860	0.424
L12	87.0	7.787	0.400
L13	94.0	9.680	0.429
L14	81.0	8.567	0.435
L15	84.0	8.429	0.424
L16	90.5	10.256	0.450
L17	73.0	7.393	0.426
L19	71.0	8.798	0.471
L20	27.5	25.829	1.296
L23	75.0	12.842	0.553
L24	79.0	9.418	0.462
L25	88.5	10.422	0.459
L27	31.0	17.422	1.002
L29	83.0	8.518	0.428
L30	112.0	13.527	0.465
L32	74.0	8.224	0.446
L33	19.5	23.271	1.461
L34	22.0	21.236	1.314
L35	25.0	24.044	1.311
L38	70.0	7.738	0.445
L39	62.0	6.538	0.434
L40	86.0	8.960	0.432
L41	34.5	16.278	0.919
L42	74.0	7.209	0.417
Plot0008_01_Aq1_ N-1	36.0	18.144	0.986
Plot0008_04_Aq1_N-1	24.0	12.351	0.951
Plot0008_06_Aq1_N-1	24.0	18.377	1.239
Plot0008_07_Aq1_ N-1	18.0	13.347	1.243
Plot0008_11_Aq1_N N-1	51.0	13.140	0.724
Plot0008_17_Aq2_N-1	36.0	15.186	0.946
Plot0008_28_Aq3_N N-3	43.0	17.061	0.964
Plot0008_44_Aq5_N	48.0	14.527	0.798
Plot0008_45_Aq5_N-1	34.0	16.358	0.998
Plot0008_46_Aq5_N N-1	21.0	12.142	1.116
Plot0008_47_Aq5_N-1	22.0	7.597	0.859
Plot0008_48_Aq5_N N-1	30.0	18.420	1.186

Plot0008_49_Aq5_N N-1	22.0	12.543	1.061
Plot0008_50_Aq5_N-1	24.0	14.763	1.166
Plot0008_51_Aq5_N N-1	31.0	11.518	0.959
Plot0008_52_Aq5_N N-1	35.0	15.921	0.993
Aq1 21_01 F-1	39.0	15.992	0.912
Aq1 21_02 F-1	45.0	18.097	0.950
Aq1 21_03 F-1	35.0	16.190	0.961
Aq1 21_04 F-1	49.0	18.399	0.892
Aq1 21_05 F-1	53.0	16.491	0.820
Aq1 21_09 F-1	57.0	15.976	0.750
Aq1 21_10 F-1	55.0	15.930	0.793
Aq1 21_11 F-1	47.0	15.680	0.897
Aq1 21_12 F-1	50.0	14.193	0.757
Aq1 21_13 F-1	33.0	16.331	1.006
Aq1 21_14 F-1	36.0	19.706	1.068
Aq1 21_15 F-1	45.0	18.339	0.943
Aq2 21_17 F-1	58.0	15.859	0.758
Aq2 21_18 F-1	40.0	18.637	1.039
Aq2 21_19 F-1	41.0	15.335	0.893
Aq2 21_21 F-1	54.0	25.060	0.984
Aq2 21_22 F-1	52.0	20.849	0.912
Aq2 21_24 F-1	59.0	18.632	0.858
Aq2 21_26 F-1	51.0	15.645	0.786
Aq2 21_27 F-1	40.0	15.314	0.950
Aq2 21_29 F-1	43.0	16.776	0.941
Aq3 21_34 F-1	38.0	12.622	0.855
Aq3 21_36 F-1	36.0	20.477	1.116
Aq3 21_37 F-1	48.0	18.051	0.918
Aq3 21_38 F-1	39.0	16.773	0.941
Aq3 21_39 F-1	61.0	21.305	0.846
Aq3 21_42 F-1	55.0	18.670	0.854
Aq4 21_45 F-1	49.0	12.174	0.744
Aq4 21_46 F-1	46.0	15.542	0.857
Aq4 21_47 F-1	46.0	18.447	0.916
Aq4 21_48 F-1	48.0	20.051	0.910
Aq4 21_49 F-1	29.0	16.516	1.068
Aq4 21_50 F-1	42.0	19.354	0.996
Aq4 21_51 F-1	50.0	15.411	0.793
Aq4 21_52 F-1	48.0	15.520	0.874
Aq4 21_53 F-1	41.0	14.386	0.853
Aq4 21_54 F-1	45.0	21.440	0.981
Aq4 21_55 F-3	39.0	17.389	0.973
Aq4 21_58 F-1	41.0	17.501	0.915
Aq1 25_42 F-2	42.0	17.362	0.958
Aq1 25_43 F-1	35.0	17.631	1.054
Aq1 25_44 F-1	46.0	14.690	0.828
Aq1 25_49 F F-1	46.0	13.037	0.775
Aq1 25_50 F F-1	34.0	16.071	1.034
Aq1 25_51 F F-1	37.0	12.763	0.846
Aq1 25_52 F-1	31.0	17.704	1.075

Aq1 25_53 F-1	45.0	14.483	0.868
Aq1 25_54 F-1	34.0	20.404	1.096
Aq1 25_55 F-1	29.0	10.967	0.897
Aq 5 26_40 F-1	44.0	13.721	0.822
Aq 5 26_41 F-1	55.0	13.078	0.711
Aq 5 26_42 F-1	47.0	14.584	0.804
Aq 5 26_43 F-1	56.0	12.595	0.679
Aq 5 26_44 F-1	44.0	9.051	0.742
Aq 5 26_45 F-1	47.0	14.956	0.825
Aq 5 26_46 F-1	47.0	22.908	0.990
Aq 5 26_47 F-1	57.0	14.236	0.734
Aq 5 26_49 F-1	33.0	11.181	0.818
Aq 5 26_50 F-1	36.0	12.391	0.861
Aq 5 26_51 F-1	43.0	13.482	0.798
Aq 5 26_52 F-1	29.0	14.516	1.011
Aq 5 26_53 F-1	48.0	14.608	0.815
Aq1 25_02 F-2	31.0	21.958	1.231
Aq1 25_03 F-2	40.0	21.660	1.047
Aq1 25_05 F-1	35.0	23.538	1.218
